# Distribution models for koalas in South Australia using citizen science-collected data

**DOI:** 10.1002/ece3.1094

**Published:** 2014-04-28

**Authors:** Ana M M Sequeira, Philip E J Roetman, Christopher B Daniels, Andrew K Baker, Corey J A Bradshaw

**Affiliations:** 1 The Environment Institute and School of Earth and Environmental Sciences, The University of AdelaideAdelaide, South Australia, 5005, Australia; 2 Barbara Hardy Institute, University of South AustraliaGPO Box 2471, Adelaide, South Australia, 5001, Australia; 3 CSIRO Land and WaterPrivate Bag No. 2, Glen Osmond, South Australia, 5064, Australia

**Keywords:** Citizen science, climate, ecological niche models, pseudo-absence, species distribution models, temperature limitation, vegetation

## Abstract

The koala (*Phascolarctos cinereus*) occurs in the eucalypt forests of eastern and southern Australia and is currently threatened by habitat fragmentation, climate change, sexually transmitted diseases, and low genetic variability throughout most of its range. Using data collected during the *Great Koala Count* (a 1-day citizen science project in the state of South Australia), we developed generalized linear mixed-effects models to predict habitat suitability across South Australia accounting for potential errors associated with the dataset. We derived spatial environmental predictors for vegetation (based on dominant species of *Eucalyptus* or other vegetation), topographic water features, rain, elevation, and temperature range. We also included predictors accounting for human disturbance based on transport infrastructure (sealed and unsealed roads). We generated random pseudo-absences to account for the high prevalence bias typical of citizen-collected data. We accounted for biased sampling effort along sealed and unsealed roads by including an offset for distance to transport infrastructures. The model with the highest statistical support (*w*AIC_*c*_ ∼ 1) included all variables except rain, which was highly correlated with elevation. The same model also explained the highest deviance (61.6%), resulted in high *R*^2^(*m*) (76.4) and *R*^2^(*c*) (81.0), and had a good performance according to Cohen's *κ* (0.46). Cross-validation error was low (∼ 0.1). Temperature range, elevation, and rain were the best predictors of koala occurrence. Our models predict high habitat suitability in Kangaroo Island, along the Mount Lofty Ranges, and at the tips of the Eyre, Yorke and Fleurieu Peninsulas. In the highest-density region (5576 km^2^) of the Adelaide–Mount Lofty Ranges, a density–suitability relationship predicts a population of 113,704 (95% confidence interval: 27,685–199,723; average density = 5.0–35.8 km^−2^). We demonstrate the power of citizen science data for predicting species' distributions provided that the statistical approaches applied account for some uncertainties and potential biases. A future improvement to citizen science surveys to provide better data on search effort is that smartphone apps could be activated at the start of the search. The results of our models provide preliminary ranges of habitat suitability and population size for a species for which previous data have been difficult or impossible to gather otherwise.

## Introduction

Predicting the spatial distribution of species can assist management strategies for wildlife by estimating *inter alia* relative densities, range sizes, and regions of potential human conflict (e.g., Hirzel et al. 2006; Elith and Leathwick 2009; Barbosa et al. 2010). In terrestrial systems where urban development is increasingly fragmenting wildlife habitat (Hanski 2011), species distribution models (resource selection functions) are particularly useful for identifying important refuge habitats. However, obtaining data necessary to develop such models can be difficult, time-consuming, and expensive, especially for nocturnal, cryptic, or wide-ranging species (Stein and Ettema 2003). To maximize the effectiveness of the typically limited funding (Caughlan and Oakley 2001; Hutchins et al. 2009) available for wildlife management, expansive, efficiently collected, reliable, and inexpensive monitoring data are essential (Lovett et al. 2007).

In select circumstances, employing the power of citizen science can provide such cost-efficient data to augment research by professional scientists (Bonney et al. 2009; Dickinson et al. 2010). Such projects involve the wider community, typically through volunteers actively collecting data, which enables researchers to increase the spatial or temporal coverage of their sampling (Dickinson et al. 2012). For mainly these reasons, citizen-collected data useful for scientific enquiry are rapidly proliferating (Cohn 2008; Couvet et al. 2008; Silvertown 2009; Roy et al. 2012). The endeavor has been facilitated more recently by new technologies, such as smartphones and Web applications that enhance the collection and quality of timely, accurate, and verifiable data (e.g., photographs and spatial coordinates) (Dickinson et al. 2012; Roy et al. 2012).

Despite the rising popularity, applications for, and quality of citizen science, there are still many idiosyncrasies in the data collected by nonprofessionals. For example, even the relatively simple category of species' presences required for habitat suitability models are subject to many potential errors, including biased sampling (nonrandom site selection), incorrect species identification, data entry errors, and a hesitancy to collect “absence” data (Cooper et al. 2012; Fink and Hochachka 2012). Carefully considering the end application of citizen-collected data, as well as limiting the opportunity for entry and identification errors via well-crafted Web applications, can minimize these biases or more easily identify their magnitude.

**Figure 1:**
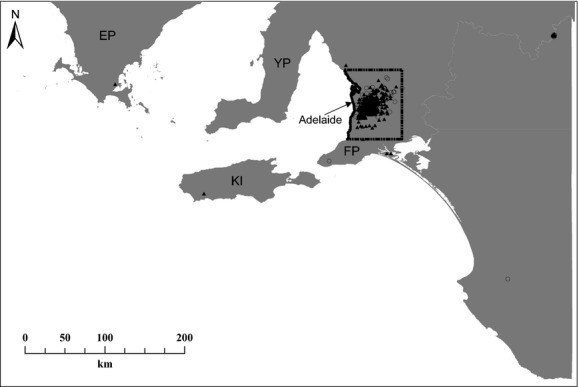
Locations with reported koala presences (black triangles) and absences (black circles) during the census within the area used for predicting koala habitat suitability in South Australia. The data used for model training are within the area covered by the dashed line. Abbreviations stand for Eyre Peninsula (EP), Yorke Peninsula (YP), Fleurieu Peninsula (FP), and Kangaroo Island (KI). The Mount Lofty Ranges are mostly within the area covered by the dashed line.

The *Great Koala Count*, a citizen science project organized in South Australia in November 2012 (koalacount.ala.org.au), was designed to gather data on the koala (*Phascolarctos cinereus*) population (including presences and absences), and the attitudes of the community toward koalas and their management. The koala is a large, wide-ranging folivore marsupial specializing on *Eucalyptus* (Moore et al. 2005) and is native in four Australian states: Queensland, New South Wales, Victoria, and South Australia. However, they were extirpated from the small southeastern corner where they occurred in South Australia following the fur trade in 1920 (Masters et al. 2004). Koalas were otherwise absent from South Australia, with no previous record of their presence since the Late Pleistocene (Hope et al. 1977; McDowell 2013). After their local extirpation, approximately 18 koalas were translocated from Victoria to Kangaroo Island, South Australia (Fig. 1) (Masters et al. 2004; Duke and Masters 2005), where lacking competition or predation (Moore and Foley 2000), they became “overabundant” (estimated population size > 5000 in 1994 and revised to 27,000 in 2001) (Masters et al. 2004). Due to their extensive eucalyptus browsing, a koala control program was started in 1997, leading to the reintroduction of 1105 koalas to the southeastern corner of South Australia (Masters et al. 2004) where they persist today. Currently, koalas are found throughout the Mount Lofty Ranges and the eastern and central suburbs of the City of Adelaide.

Despite their introduction success in South Australia, koala populations are declining in Australia's eastern states (NSW Department of Environment and Conservation 2006; Queensland Government 2009) and the species is listed nationally as *Vulnerable* under the Environment Protection and Biodiversity Conservation Act 1999 (http://www.environment.gov.au/topics/about-us/legislation/environment-protection-and-biodiversity-conservation-act-1999). Declines are attributed to multiple threats (Rhodes et al. 2011) such as habitat fragmentation from urban development (Melzer et al. 2000; Seabrook et al. 2003), vehicle collisions (Dique et al. 2003), and disease (Polkinghorne et al. 2013). Even where populations are stable or increasing, genetic diversity is low (Seymour et al. 2001) and is possibly causing inbreeding depression (Cristescu et al. 2009; Whisson and Carlyon 2010).

The status, abundance, and range of the koala population reintroduced to mainland South Australia have not yet been investigated. Here, we use the presence and absence data collected during the *Great Koala Count* to predict suitable habitat in South Australia (including Kangaroo Island and the mainland regions where they have been reintroduced). Our main aim is to (1) provide state-of-the-art statistical approaches to account for potential biases arising from citizen surveys, (2) assess habitat suitability for the species in South Australia accounting for both environmental conditions and anthropogenic impacts, and (3) provide a preliminary population estimate based on suitability and plausible densities. Our work shows that citizen science can be an effective means to collect meaningful scientific data and that their usefulness can be enhanced if appropriate statistical tools are applied.

## Materials and Methods

### Koala presence/absence data

Koala occurrence data were collected during the *Great Koala Count* held on 28 November 2012 mainly in Adelaide and the Mount Lofty Ranges region of South Australia. The project was promoted to the wider community via (1) a partnership with a local radio station (891 ABC Adelaide), (2) a specifically developed education project for schools, (3) social media (particularly via Facebook), (4) incidental media coverage (television, radio, and newspaper), and (5) the communication networks of the project partners (see Acknowledgments). We asked volunteer “citizen scientists” to search for koalas between 06.00 and 20.00 on the day of the count and to report both sightings and nonsightings (i.e., presences and absences). A live radio broadcast on the morning of the count promoted participation through discussion about koalas in South Australia and how the public could collect and submit data. Reports could be made through the *Great Koala Count* website (koalacount.ala.org.au), or in near-real time via Apple® and Android® smartphone apps adapted from existing mobile applications created to feed citizen-science data to the *Atlas of Living Australia* (ALA) website (ala.org.au).

Data reported included (1) location (via GPS for smartphones and online mapping tools for the website), (2) a photograph for validation of the sighting, (3) search effort in number of minutes, and (4) information about the activity of the observed koala(s) (e.g., *sleeping*, *sitting*, *eating*, *climbing*, *drinking*, *walking*, *dead*, or *other*). Data were integrated with the Biological Data Recording System and stored in a bespoke web portal, hosted by the ALA. Submitted records were visualized in near-real time and displayed on the project website. We checked all records prior to inclusion in our model, removing duplicates or obviously erroneous entries (e.g., other species). We contacted citizen scientists via e-mail for clarification if their records were suspected to be inaccurate. If inaccuracies could not be rectified, we removed the associated records. The cleaned dataset included 1359 reports with exact location (latitude and longitude in WGS84 coordinates with a precision of 0.01°) counting 1244 koala sightings (i.e., presences) and 115 absences. The data mostly covered the metropolitan area of Adelaide (the main city in South Australia), and most reported koalas were sighted nearby within conservation parks in the Adelaide Hills or along streets or in backyards of the Hills towns and Adelaide suburbs.

Following Rhodes et al.' (2009) suggestion that koala occurrence is best described by habitat variables measured at a 1-km^2^ resolution, we overlayed a 1-km^2^ grid cell on the ∼ 5576-km^2^ main area covered by the census (Fig. 1). The presence/absence reports resulted in a total of 255 “presence” grid cells and 32 absences. Due to the low number of reported absences, we randomly selected 10 pseudo-absences for each presence (Barbet-Massin et al. 2012). A random selection of pseudo-absences yields the most reliable distribution models when using regression techniques (Barbet-Massin et al. 2012). We down-weighted the pseudo-absence grid cells to 0.1, and conversely, we weighted grid cells with reported sightings/absences to one or more (whenever >1 report fell within the same grid cell). To account for model bias due to the location of pseudo-absences selected, we iterated the pseudo-absence selection process 100 times (Sequeira et al. 2013).

### Environmental data and anthropogenic disturbances

We obtained spatial data on vegetation, topographic water features, transport infrastructure (sealed and unsealed roads), and elevation from the Department of Environment, Water and Natural Resources (DEWNR), Government of South Australia. From the Australian Government Bureau of Meteorology (http://www.bom.gov.au), we obtained 20-year monthly averages (from 1993 to 2012) of maximum and minimum temperature, water vapor pressure, solar exposure (with no data for November 2009), and rainfall.

We assembled the vegetation data based on the dominant tree species and considering three main groups: (1) “koala eucalyptus”: *Eucalyptus* species mostly used (eaten and sheltered in) by koalas in South Australia (manna gum *E. viminalis*, blue gum *E. leucoxylon*, red gum *E. camaldulensis,* and stringybark *E. baxteri*, *E. obliqua,* and *E. macrohyncha*) (Masters et al. 2004); (2) “other eucalyptus”: other *Eucalyptus* species present in South Australia but not commonly used by koalas; and (3) “other vegetation” (i.e., not *Eucalyptus*). We used the areas of each dominant group within each grid cell as predictors in our models. We included topographic water features by considering distance to inundated areas as proxy for drinking water availability, considering only year-round and seasonally inundated areas (water bodies). We also included the density of watercourses (i.e., rain watercourses) within each grid cell as a proxy for food quality (i.e., possibly reflecting leaf water content). We interpolated the elevation data using inverse-distance-weighted interpolation and extracted values from the resulting surface to each grid cell.

To account for anthropogenic disturbances, we included two groups of road density: (1) sealed roads, representing areas with high anthropogenic pressure, and (2) unsealed roads (e.g., park paths), representing lower potential disturbance. To calculate distances, we used the *Near* tool in ArcGIS™ 9.3.1 (ESRI, Redlands, CA) with an equidistant cylindrical projection. We estimated areas with the function *calculate geometry* using a Mollweide (equal area) projection. For road densities, we summed the length of sealed and unsealed roads within each grid cell.

Our predictor dataset (after excluding collinear variables) included the following for each grid cell: (1) the area with “koala eucalyptus” (*koalaeuc*; m^2^), (2) the area with “other eucalyptus” (*othereuc*; m^2^), (3) the area with trees other than eucalyptus (*otherveg*; m^2^), (4) the density of watercourses (*denswater*; m of water course per 1 km^2^), (5) the distance to water bodies (*dist2water*; m), (6) elevation (*elevation*; m), (7) the density of sealed roads (*sealed*; m of road per 1 km^2^), (8) the density of unsealed roads (*unsealed*, m of road per 1 km^2^), (9) the temperature range for the month of November calculated as the difference between the average maximum and minimum temperatures (*temp.range*; °C), and (10) the average rainfall for the month of November (*rain*; mm).

**Figure 2:**
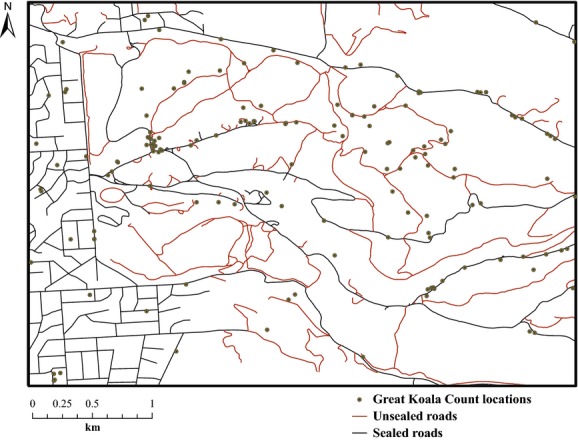
Detail showing locations reported during the census. Most of the locations occur near sealed roads and park paths (unsealed roads).

Citizen-collected data were highly biased toward unsealed and sealed roads (Fig. 2); therefore, we used the inverse of distance to the closest road (whether sealed or unsealed) as an offset in the models to account for this sighting effort bias. Together with the weighting considered between reported locations and pseudo-absences, we considered this offset a proxy for sampling effort. We assessed collinearity between predictors using Spearman's *ρ* within the *pairs.panels* function from the *psych* package in R (Revelle 2013).

### Model development and spatial autocorrelation

Using the koala presence/absence data in each grid cell as a response, we first developed generalized linear models (GLM) with a binomial distribution (logit link function) with ten predictors for the complete dataset. The model set included several possible combinations of these predictors (i.e., explanatory variables or fixed effects) as well as an offset for our proxy of sampling effort (Table 1). To stabilize parameter estimation, we centered the explanatory variables included in each model. Following Burnham and Anderson (2004), we used Akaike's information criterion corrected for small sample sizes (AIC_*c*_) to rank models. We then calculated the relative model weights (*w*AIC_*c*_) to assess the model strength of evidence.

**Table 1:** Summary of generalized linear mixed-effects models relating probability of koala suitable habitat as a function of environment and climatology in South Australia. Predictors include the following: *veg* (all vegetation variables, i.e., koala *Eucalyptus – koalaeuc*, other *Eucalyptus* – *othereuc,* and other vegetation); *water* (density of watercourses and distance to water bodies), *temp.range* (temperature range), *elevation*, *roads* (density of sealed and unsealed roads), and *rain*. *Null* represents the null model (i.e., without fixed effects). All models included an offset term for effort, and the generalized linear mixed-effects models also included a spatial random effect (2-km^2^ grid cell). Shown for each model are biased-corrected model probabilities based on weights of Akaike's information criterion corrected for small sample sizes (*w*AIC_*c*_, only >0.001 shown), Bayesian information criteria (*w*BIC, only >0.001 shown), the percentage of deviance explained (%De), the cross-validation error (CV_error_), and Cohen's *κ* and both marginal and conditional *R*^2^ (*R*^2^(*m*) and *R*^2^(*c*); Nakagawa and Schielzeth (2013)

Model	*w*AIC_*c*_	*wB*IC	%De	CV_error_	*κ*	*R*^2^(*m*)	*R*^2^(*c*)
*veg* + *water* + *temp.range* + *elevation* + *roads*	∼1	1	61.6	0.10 ± 0.01	0.46 ± 0.07	76.4	81.0
*veg* + *water* + *temp.range* + *rain* + *roads*	<0.001	<0.001	57.6	0.13 ± 0.01	0.33 ± 0.06	66.2	72.8
*veg* + *water* + *temp.range* + *elevation*	<0.001	<0.001	57.0	0.12 ± 0.02	0.39 ± 0.07	67.5	73.8
*veg* + *temp.range* + *rain*	<0.001	<0.001	53.7	0.12 ± 0.01	0.40 ± 0.07	59.9	71.7
*veg* + *water* + *temp.range* + *rain*	<0.001	<0.001	53.9	0.13 ± 0.01	0.31 ± 0.06	60.4	68.1
*temp.range* + *rain*	<0.001	<0.001	52.3	0.10 ± 0.02	0.27 ± 0.09	56.2	76.7
*veg* + *temp.range*	<0.001	<0.001	48.2	0.14 ± 0.02	0.30 ± 0.06	52.9	62.1
*temp.range*	<0.001	<0.001	45.0	0.11 ± 0.03	0.05 ± 0.04	36.2	77.2
*elevation*	<0.001	<0.001	41.4	0.15 ± 0.02	0.24 ± 0.08	5.0	81.6
*veg* + *water*	<0.001	<0.001	15.3	0.15 ± 0.02	0.24 ± 0.08	27.5	41.6
*veg*	<0.001	<0.001	14.9	0.11 ± 0.02	0.04 ± 0.04	26.3	40.6
*koalaeuc*	<0.001	<0.001	11.6	0.16 ± 0.03	0.20 ± 0.07	22.1	37.2
*roads*	<0.001	<0.001	1.8	0.11 ± 0.03	0.04 ± 0.04	17.0	33.1
*null*	<0.001	<0.001	–	0.16 ± 0.02	0.14 ± 0.07	0.0	0.0
*water*	<0.001	<0.001	5.7	0.16 ± 0.02	0.04 ± 0.06	4.0	22.7

We assessed the expected spatial autocorrelation (McAlpine et al. 2008; Rhodes et al. 2009) both in the observations and in model residuals by calculating Moran's *I* (Diggle and Ribeiro 2007) after a Bonferroni correction (Legendre and Legendre 1998). For this, we used the *sp.correlogram* function from the *spdep* library in R (Bivand 2013). To reduce the spatial autocorrelation observed in the residuals of the GLM, we included a spatial random effect for grid cell size (4 km^2^) in the model set (Lunney et al. 2009; Rhodes et al. 2009). Including this random effect leads to the development of generalized linear mixed model (GLMM) with the *lmer* function from the *lme4* package in R (Bates et al. 2013). We used the marginal and conditional *R*^2^ (Nakagawa and Schielzeth 2013) alongside the percentage of deviance explained (%De) by each model as indices of goodness of fit. We assessed the model's predictive power using Cohen's *κ* (Cohen 1960), and we also used a 10-fold cross-validation (Davison and Hinkley 1997) to calculate the mean prediction error for the highest-ranked model. We report the median model rankings obtained from the 100 pseudo-absence iterations.

We calculated the effect sizes for each predictor by (1) dividing the coefficient estimates of each predictor by their standard error (Bradshaw et al. 2012), (2) calculating the fraction of the relative model weight (*w*AIC_*c*_) from the sum of weights where each predictor occurred and then (3) multiplying the standardized predictor coefficient estimates (accounting for the standard error) by the fraction of the relative model weight, and (4) summing each predictor contribution for all models where they occurred. We developed all models using R (R Core Team 2013).

### Estimating abundance

There is usually a disconnect between predicted environmental suitability and species abundance (Murphy et al. 2006; Sagarin et al. 2006), such that indices of habitat quality cannot necessarily be used directly to infer relative abundance. Fortunately, recent empirical evidence for 69 Australian vertebrates demonstrates that for most species, there is a linear or curvilinear relationship between habitat suitability inferred from species distribution models and relative abundance (VanDerWal et al. 2009). With no specific information available for koalas, we assumed a simple linear relationship between our relative habitat suitability index (0 = lowest; 1 = highest) and density following the assumption that the relationship increased linearly up to half maximum density (D_*max*_) (see Fig. 2 in VanDerWal et al. 2009). As an estimate of D_*max*_, we took the mean of nine published upper-level density estimates for populations across Australia (excluding extremely high density values >4 ha^−1^; Table S1); this gave 1.57 ha^−1^ (SD = 1.19) or 157 ± 119 km^−2^ (Table S1). Summing over all 1-km^2^ grid cells provides an estimate of the total population size within the 5576-km^2^ Adelaide–Mount Lofty ranges study area.

## Results

The predictor variables showed no evidence of major collinearity (all Spearman's *ρ* < 0.5) except for *elevation* and *rain* (*ρ* = 0.90). For this reason, we did not include these two predictors together in the same model. Moran's *I* correlograms showed high spatial autocorrelation in the residuals of the GLM (*p* < 0.001; Fig. S1). Including the spatial random effect in the GLMM assisted in reducing the spatial autocorrelation to close to zero (Fig. S1), and therefore, we only provide the GLMM results.

The model with all predictors except *rain* had the highest statistical support (*w*AIC_*c*_ > 0.999), and both highest *R*^2^(*m*) (76.) and deviance explained (>61%) (Table 1). *R*^2^(*c*) for this model was the second highest (81.0). *Elevation* alone received a slightly higher *R*^2^(*c*) (81.6) and explained 41.4% of the deviance. While the model including only the anthropogenic disturbances (i.e., densities of sealed and unsealed roads) explained < 2% of the deviance, by adding these variables to other predictors, the deviance explained increased by ∼ 4.5% (Table 1). The cross-validation error was ∼ 0.1, and *κ* was 0.46 ± 0.07 for the top-ranked GLMM (Table 1).

**Figure 3:**
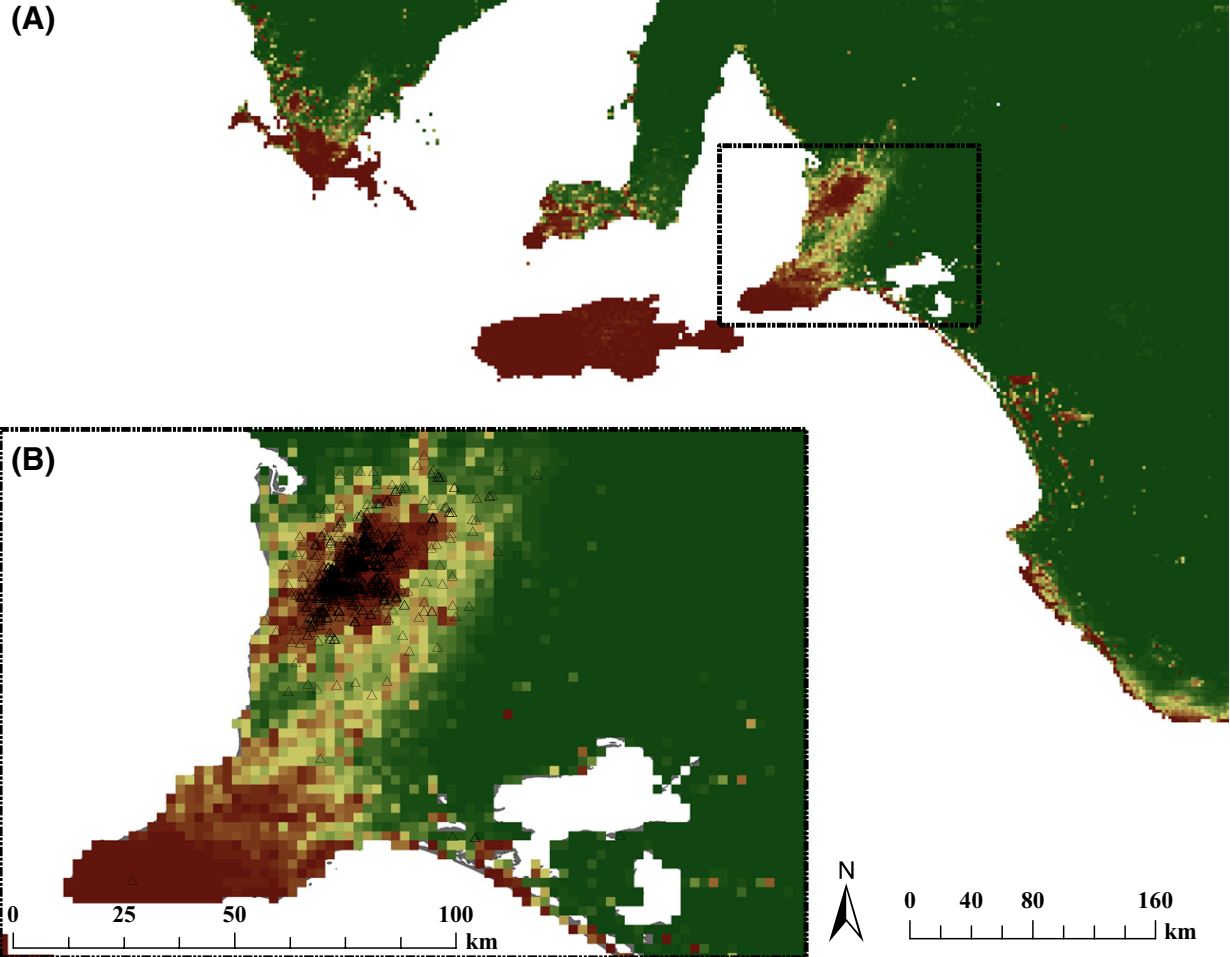
Predicted habitat suitability for koalas in South Australia derived from the generalized linear mixed-effects models: (A) prediction to the southern part of South Australia, including areas where koalas could occur; (B) area covered by the census (5531 km^2^) and used to train the models; and black triangles represent reported koala presences.

The models revealed highest habitat suitability for koala occurrence in the region of the Adelaide–Mount Lofty Ranges where most of the reported presences were obtained (Fig. 3). When predicting to the southern section of South Australia, habitat suitability was estimated >0.9 in Kangaroo Island. Similar suitability was obtained for the southern extremities of the three South Australian peninsulas (Eyre, Yorke, and Fleurieu Peninsulas) and in scattered locations along the southeastern coast of South Australia (Fig. 3).

**Figure 4:**
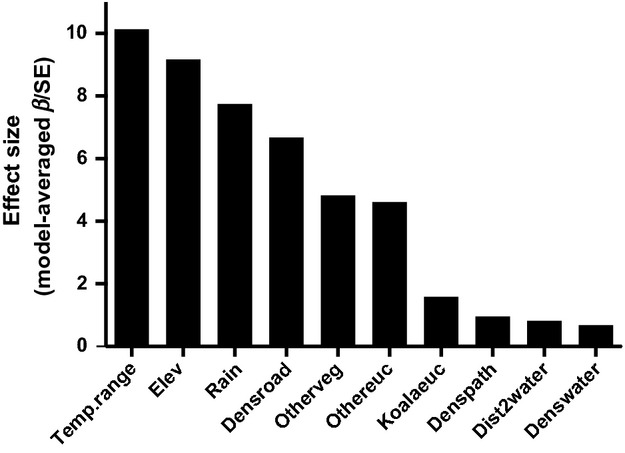
Model-averaged effect sizes calculated for each predictor included in the generalized linear mixed-effects models: *denswater* – density of watercourses, *dist2water* – distance to water bodies, *denspath* – density of unsealed roads, *koalaeuc –* koala *Eucalyptus*, *othereuc* – other *Eucalyptus*, *otherveg –* other vegetation, *densroad* – density of sealed roads, *rain,* elev – elevation, and *temp.range* – temperature range.

**Figure 5:**
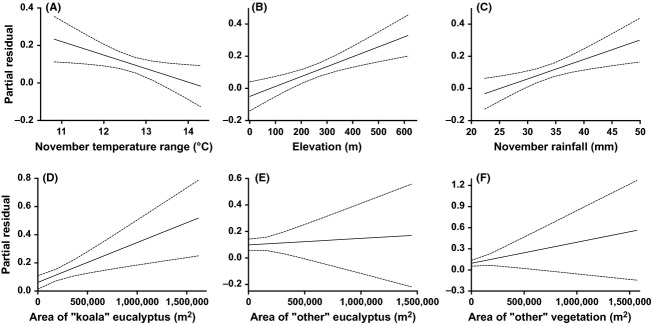
Partial residual plots for the climate and vegetation fixed effects included in the top-ranked models: (A) November temperature range (°C), (B) elevation (m), (C) November rainfall (mm) (from second-most highly ranked model), (D) area of “koala” eucalyptus (m km^−2^), (E) area of “other” eucalyptus (m km^−2^), and (F) area of “other” vegetation (m km^−2^).

**Figure 6:**
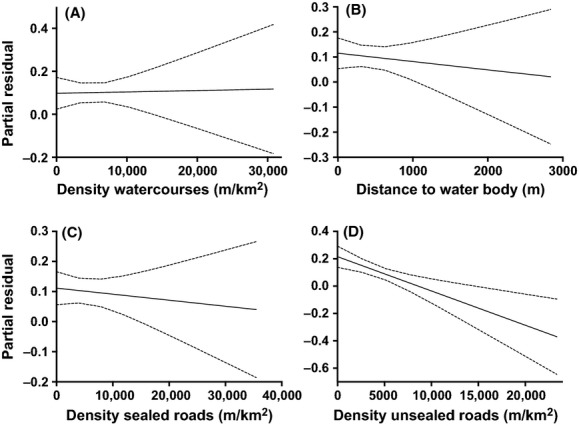
Partial residual plots for the water and roads fixed effects included in the top-ranked models: (A) density of watercourses (m km^−2^), (B) distance to water bodies (m), (C) density of sealed roads (m km^−2^), and (D) density of unsealed roads (m km^−2^).

According to the model-weighted, standardized parameter estimates, temperature range had the highest contribution (effect size = 10.13) in habitat suitability, followed by elevation, rainfall, density of sealed roads, and vegetation (Fig. 4). Temperature range had the largest negative effect on koala occurrence (Figs. 4 and 5A), reflecting that koalas use habitats with relatively more constant temperatures. Higher relative elevation and rainfall correlated positively with koala occurrence (Fig. 5B and C). Density of unsealed roads, distance to water bodies, and density of watercourses all had a contribution <1 (Fig. 6).

Based on the linear relationship between density and habitat suitability, we estimated the mean total population in the Adelaide–Mount Lofty Ranges study area (5576 km^2^) at 113,704 (95% confidence interval based on ±2 standard deviations of maximum density: 27,685–199,723). This equates to an average density over the study area of 5.0–35.8 km^−2^. The large uncertainty is a function of the high coefficient of variation (100 × 1.19/1.57 = 75.5%) of the maximum density estimate applied.

To understand whether our models were successful in dealing with the bias in our dataset, we compared the results of the top-ranked model with results when disregarding some of the components added to deal with biases (Table 2). This comparison resulted in poorer deviance explained and poorer goodness of fit (despite the higher *R*^2^) when not considering components associated with the presence/absence bias, but no considerable change when removing the offset term (only slightly lower *R*^2^). These results indicate that a larger offset should possibly have been considered. However, a test run specifically considering a larger contribution for the offset term (not shown) resulted in a prediction of zero koala occurrence in South Australia.

**Table 2:** Summary of the generalized linear mixed-effects model results disregarding some of the components used to deal with biases associated with citizen-collected data. Only results for the highest-ranked model (*veg* + *water* + *temp.range* + *elevation* + *roads*) are shown. Predictors include the following: *veg* (all vegetation variables, i.e., koala *Eucalyptus – koalaeuc*, other *Eucalyptus* – *othereuc,* and other vegetation); *water* (density of watercourses and distance to water bodies), *temp.range* (temperature range), *elevation*, and *roads* (density of sealed and unsealed roads). All models included a spatial random effect (2-km^2^ grid cell). Shown for each model are biased-corrected model probabilities based on weights of Akaike's information criterion corrected for small sample sizes (*w*AIC_*c*_), the percentage of deviance explained (%De), the cross-validation error (CV_error_), and Cohen's *κ* and both marginal and conditional *R*^2^

Disregarding:	*w*AIC_*c*_	%De	CV_error_	*κ*	*R*^2^(*m*)	*R*^2^(*c*)	Largest effect
*Original*	∼1	61.6	0.10 ± 0.01	0.46 ± 0.07	76.4	81.0	*Temperature (10.13)*
*Offset*	1	62.7	0.11 ± 0.03	0.48 ± 0.07	75.9	80.6	*Temperature (10.20)*
*Weights*	1	49.0	0.25 ± 0.02	0.15 ± 0.03	82.3	83.2	*Sealed roads (21.26)*
*Pseudo-absences*^1^	1	49.0	0.01 ± <0.01	0.14 ± 0.02	82.3	83.2	*Sealed roads (21.26)*

1 The model disregarding pseudo-absences uses the entire background environmental data within the area covered by the dashed line in Fig. 1.

## Discussion

We have provided the first citizen science-generated estimates of koala habitat suitability and population size by applying state-of-the-art technology and statistical techniques to account for the typical biases and uncertainty in data collected by nonscientists (Silvertown 2009; Dickinson et al. 2010; Courter et al. 2013). The use of the photographic application on mobile phones enabled us to confirm the counted animal as a koala. GPS technology facilitated the collection of geolocated data, which enables any double counting to be detected easily. The census day was hot (35°C maximum), and koalas do not move from their trees during daylight hours, so many of the traditional sampling biases for mobile animals were avoided.

In many cases, the goodwill and effort of citizen scientists are invalidated by the error-ridden data they collect, thus making the entire exercise largely useless for scientific applications (Mayer 2010). Of course, citizen science activities serve purposes other than just scientific data collection (e.g., engagement, learning, appreciation) (Trumbull et al. 2000), but they should primarily serve to collect data that can truly advance knowledge, especially in cases where such data would be too difficult or expensive to collect otherwise. An example of a successful project involving citizen science is the study on dung decomposition by beetles (Kaartinen et al. 2013), which allowed assessment of large-scale factors (across all of Finland) affecting dung decomposition. Another good example for the amount of data that can be generated by citizen scientists and used for scientific purposes is *The Christmas Bird Count*, a long-term citizen science survey running since 1900 (http://birds.audubon.org/christmas-bird-count). In 2011 alone, the project attracted 63,227 observers who collectively counted nearly 65 million bird observations (LeBaron 2012). The data collected have been used in an extensive list of scientific publications within different themes, such as community ecology (Hurlbert and Haskell 2003), biogeography (Root 1988), and patterns of population change (Niven et al. 2004; Link et al. 2006). Koala counting is potentially an ideal citizen science project because the target species is large, common, easily visible, stationary (over a day), and popular. Further, a citizen science approach to data collection is appealing as koalas are widely distributed and the costs of a professional count would be prohibitive.

The spatial and/or temporal nonrandomness in sampling effort typical of many citizen-collected datasets (Snäll et al. 2011; Hurlbert and Liang 2012) can render their analysis challenging. While we partially dealt with the location bias by introducing an offset for distance to roads in our models (Fig. 2), timing-associated biases were dealt with prior to analysis by restricting data collection to 1 day only. This could also be applied (or extended) to other citizen data for which the timing of observations is biased (Hurlbert and Liang 2012) by (1) using only a subset of the data collected or (2) requesting data collection specifically outside the peak season when it is normally collected. While statistical approaches cannot rectify all problems, they can potentially rescue many citizen science datasets. The bias of citizen scientists toward reporting only sightings of species (rather than absences) can also be problematic. In our case, we accounted for the lack of real absences by generating pseudo-absences, but this is merely a preliminary step. We suggest that the collection of real absences should be improved in future projects.

Changing the searching protocol and allowing the smartphone apps to collect data automatically during the search could potentially provide such information. For example, participants would be required to activate their app as soon as they start searching (rather than just when they find a koala), so that the apps could report nonsightings at regular intervals (temporal or spatial) while koalas are not detected. Further advantages of this method are that it provides (1) accurate information about search effort and (2) possible improvement of detection probability (Pollock et al. 2002) by allowing comparison of sighting provided by different participants on the same tracks. Another possible way to improve information associated with sampling effort is to allow citizens to gauge their own participation. This could be done by including a question asking the citizen scientist to estimate his or her search intensity (e.g., from 1 to 10, with 1 representing a casual sighting independent of active searching, and 10 representing an extremely active search, specifically trying to obtain a sighting).

The new ecological knowledge we generated with the citizen science dataset has many potential theoretical and management applications. Our results show that in addition to the expected highly suitable habitat on Kangaroo Island (Masters et al. 2004) and in the Adelaide–Mount Lofty Ranges region itself, there are also suitable areas in the southern regions of Eyre, Yorke, and Fleurieu Peninsulas (Fig. 1). However, as is happening in other Australian states (Seabrook et al. 2003), predicted suitable habitat in mainland South Australia is highly scattered, with most suitable fragments occurring on the southeastern coast of South Australia. The predicted habitat suitability outside the area used to calibrate the model (the section of Mount Lofty range, Fig. 1) is coherent with the few scattered sightings reported elsewhere within South Australia during the *Great Koala Count*, and also with the locations where koalas were reintroduced from Kangaroo Island as part of the population control program from 1997.

Temperature range had the largest negative effect on koala occurrence, suggesting that koalas use habitats with relatively more constant temperatures. Higher relative elevation and rainfall correlated positively with koala occurrence. Together, these results suggest that deviations from ideal microclimatic conditions are the most limiting components of the physical environment for this species at the edge of its range. Given the poor predictive performance of the vegetation indices (see below), it is also plausible that climate and elevation were reasonable surrogates for food and sheltering vegetation.

The weak effect of the vegetation variables might arise in part from the biased sampling. The contribution of the koala eucalyptus vegetation class was also unexpectedly lower than the contribution from the other vegetation variables. Occurrence data were collected only during 1 day (28 November 2012), and temperatures were above 30°C during most of that day, reaching a maximum of 35.4°C. If koalas were mostly using trees as heat shelters rather than for feeding (a typical behavior on extremely hot days) (Crowther et al. 2013), the importance of certain vegetation types could have been overlooked. Indeed, <9% of the sightings reported a koala feeding, with most reporting sleeping or low activity (57% sleeping/sitting). Moreover, our vegetation classification was based on the most dominant tree species, so areas classified as “other” (i.e., dominant species other than *Eucalyptus*) could still contain *Eucalyptus* species. Also, the vegetation layers we used contained data acquired in 2000 and might not have accurately described current vegetation conditions.

Interestingly, despite the low deviance explained by density of roads alone, this variable was particularly important for predicting the occurrence of koalas. A similar result was obtained by Rhodes et al. (2006) in areas where anthropogenic impacts occurred nearby. While there is clearly much yet to discern about koala distribution and population size in South Australia, our paper demonstrates that citizen science-collected datasets can be useful to advance ecological knowledge about particular species and ecosystems. Continuing to refine the survey protocol, as well as collecting more data across different areas and climatic conditions, would increase the confidence in our predictions.

## Supporting Information

**Figure S1.** Moran's *I* plots showing the reduction in spatial autocorrelation in the GLM residuals when a random effect was included to the models (GLMM). **Figure S2.** Comparison of predicted habitat suitability for koalas in South Australia derived from the generalized linear mixed-effects models excluding: (A) weight difference between recorded and generated pseudo-absences; (B) pseudo-absences generation (i.e., using only the recorded absences); and (C) the offset to account for effort bias near roads and paths. **Table S1.** Published population density estimates for koalas. All estimates expressed in koalas ha^−1^.
