# Supplementary material for: Distribution models for koalas in South Australia using citizen science-collected data
Source: Ecol Evol. 2014 Apr 28;4(11):2103–14. doi: 10.1002/ece3.1094 (PMC4201425; doi:10.1002/ece3.1094)
Supplement: Supplementary file 1 [file ece30004-2103-sd1.docx]

**Online Supporting Information**

**Distribution models for koalas in South Australia using citizen science-collected data**

Ana M. M. Sequeira, Philip E. J. Roetman, Christopher B. Daniels, Andrew K. Baker, Corey J. A. Bradshaw

**Contents**

***Appendix 1: Supporting Results***

**1. Supporting Figures & Tables**

*(i)* Figure S1

*(ii)* Figure S2

*(ii)* Table S1

***Appendix 1: Supporting Results***

**1. Supporting figures**

# Fig. S1. Moran’s *I* plots showing the reduction in spatial autocorrelation in the GLM residuals when a random effect was included to the models (GLMM). Moran’s *I* is shown on the *y* axis and the lags on the *x* axis. See Table 1 for variables included in each model.

| GLM | 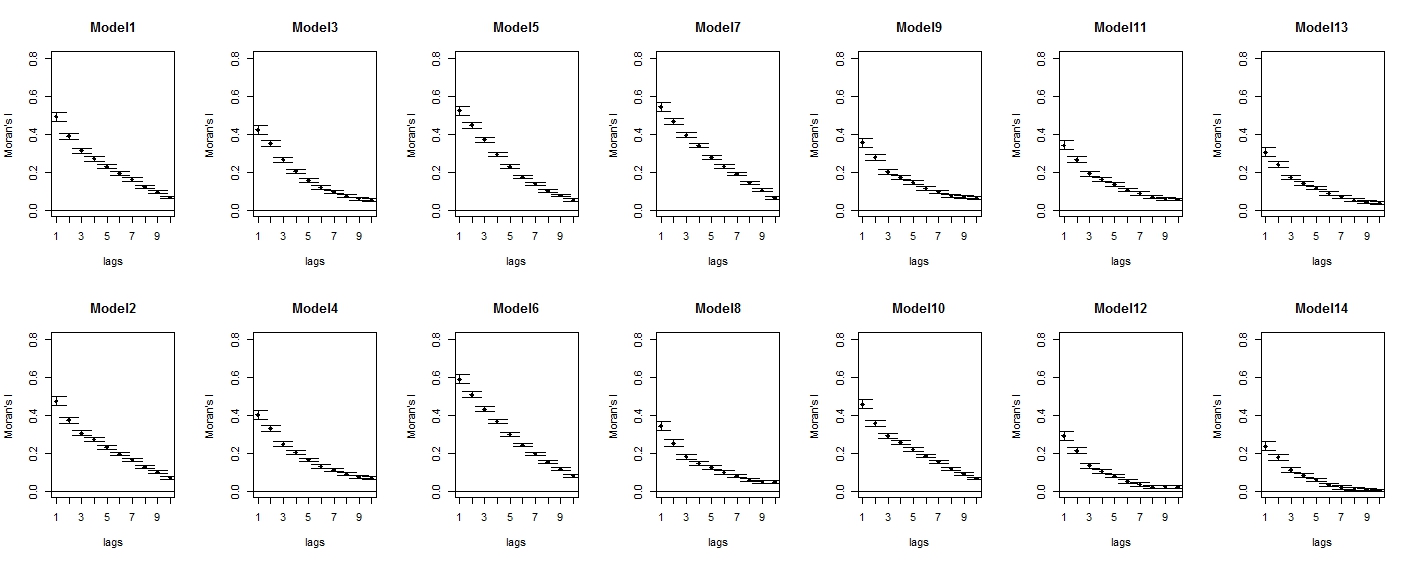 |
| --- | --- |
| GLMM | 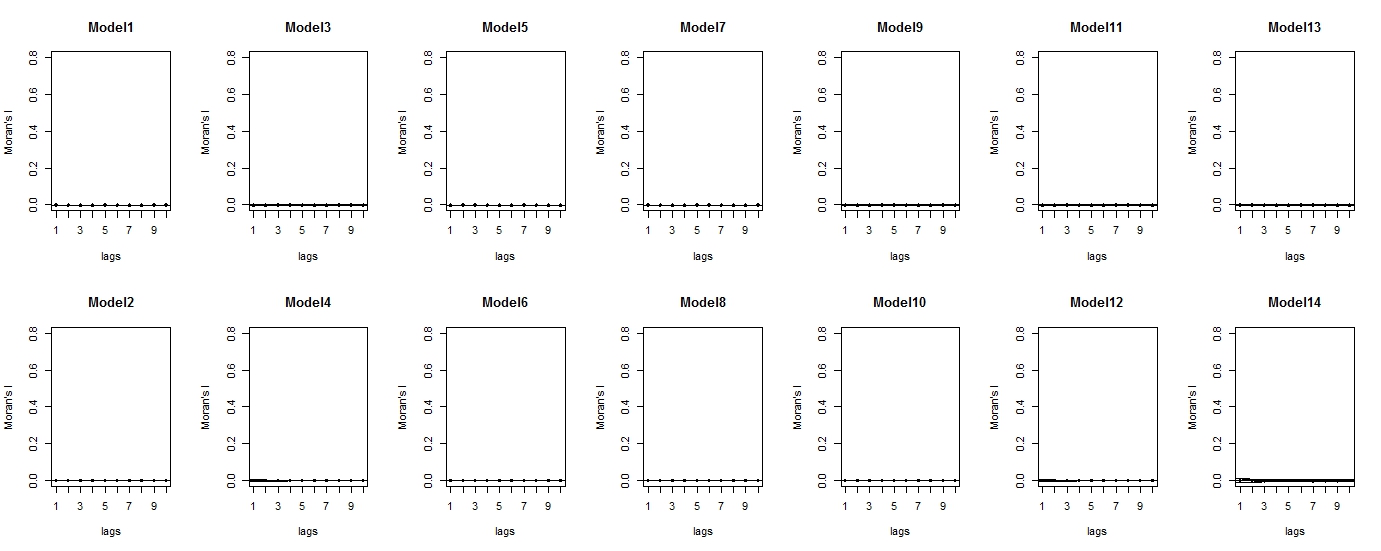 |

**Fig S2.** Comparison of predicted habitat suitability for koalas in South Australia derived from the generalized linear mixed-effects models excluding: a) weight difference between recorded and generated pseudo-absences; b) pseudo-absences generation (i.e., using only the recorded absences); and c) the offset to account for effort bias near roads and paths.

| 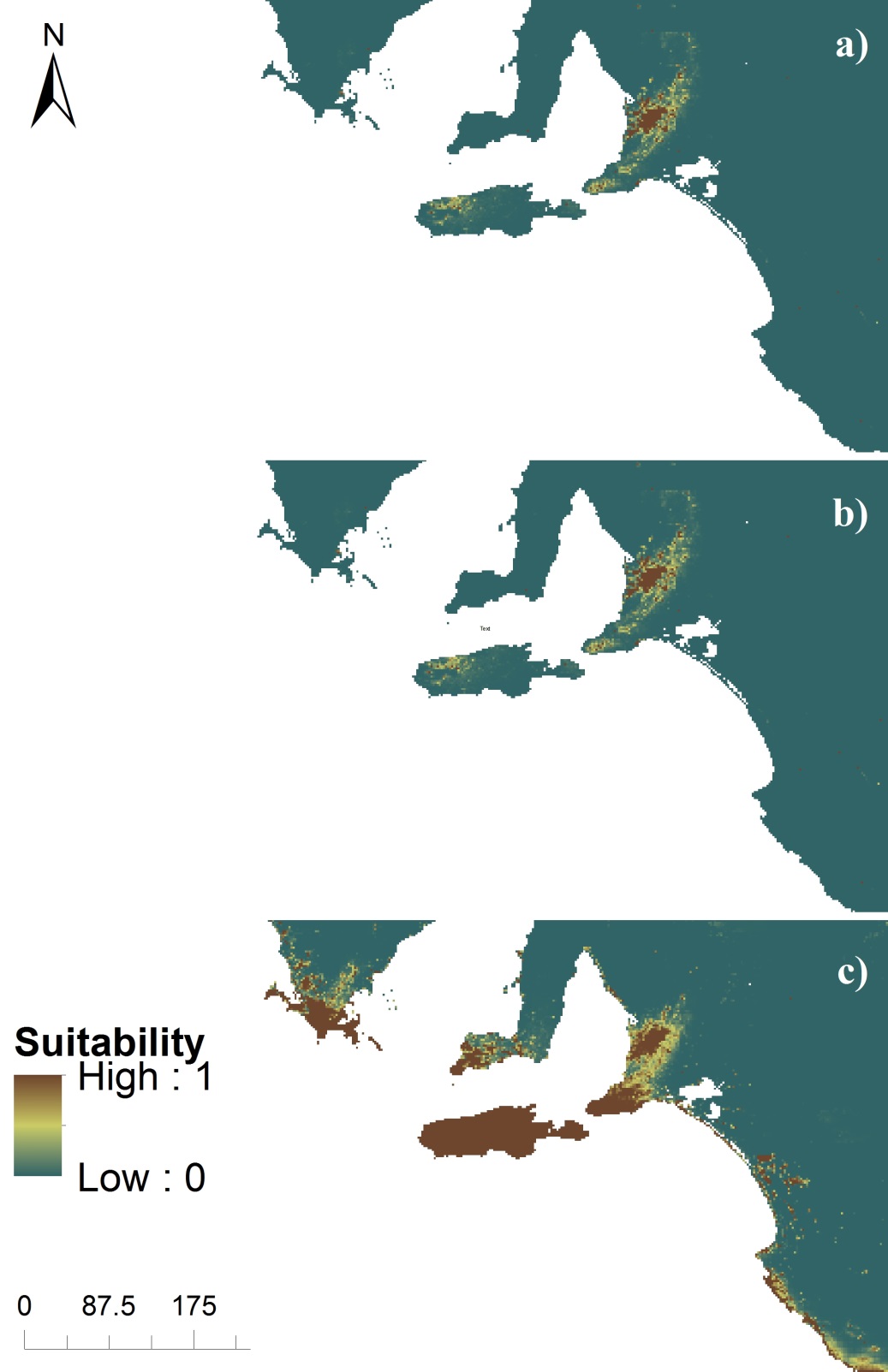 |
| --- |

**Table S1.** Published population density estimates for koalas. All estimates expressed in koalas ha^‑1^.

| lower | mid | upper | region | ref |
| --- | --- | --- | --- | --- |
| 0.0007 |  | 2.513 | SW Queensland | [[1](#_ENREF_1)] |
| 0.02 |  | 1.26 | SE Queensland | [[2](#_ENREF_2)] |
|  | 0.73 to 0.76 |  | SE Queensland | [[3](#_ENREF_3)] |
| 0 |  | 0.79 | SE Queensland (bush) | [[3](#_ENREF_3)] |
| 0 |  | 0.62 | SE Queensland (urban) | [[3](#_ENREF_3)] |
| 1 |  | 3 | Central and SE Queensland | [[4](#_ENREF_4), [5](#_ENREF_5)] |
| 0.01 |  |  | Central Queensland | [[6](#_ENREF_6)] |
| 0 |  | 0.404 | Magnetic Island, Queensland |  |
|  | 0.297 |  | mixed eucalypt forest, Magnetic Island, Queensland | [[7](#_ENREF_7)] |
| 0.7 |  | 1.6 | Brisbane Ranges, Victoria | [[8](#_ENREF_8)] |
| 6 |  | *8.9 | French Island, Victoria | [[9](#_ENREF_9)] |
| 8.62 |  | *8.85 | Strathbogie Plateau, Victoria | [[10](#_ENREF_10)] |
| 1.97 |  | 3.57 | Cygnet River Valley, Kangaroo Island, South Australia | [[11](#_ENREF_11)] |
| 0.2 |  | 0.4 | Brownhill Creek, Adelaide Hills, South Australia | [[12](#_ENREF_12)] |
|  |  | *10.9 | Flinders Chase, Kangaroo Island, South Australia | [[11](#_ENREF_11)] |
| 0.006 |  |  | Eden, New South Wales | [[13](#_ENREF_13)] |
| 4 |  | *8 | NE New South Wales | [[14](#_ENREF_14), [15](#_ENREF_15)] |
|  |  |  |  |  |
| mean (excl. high extremes) | | **1.57** |  |  |
| SD (excl. high extremes) | | **1.19** |  |  |

*Values excluded from mean calculation.

**References**

1. Sullivan B.J., Baxter G.S., Lisle A.T., Pahl L., Norris W.M. 2004 Low-density koala (*Phascolarctos cinereus*) populations in the mulgalands of south-west Queensland. IV. Abundance and conservation status. *Wildlife Research* **31**(1), 19-29. (doi:http://dx.doi.org/10.1071/WR02037).

2. Dique D.S., Preece H.J., Thompson J., de Villiers D.L. 2004 Determining the distribution and abundance of a regional koala population in south-east Queensland for conservation management. *Wildlife Research* **31**(2), 109-117. (doi:http://dx.doi.org/10.1071/WR02031).

3. Dique D.S., de Villiers D.L., Preece H.J. 2003 Evaluation of line-transect sampling for estimating koala abundance in the Pine Rivers Shire, south-east Queensland. *Wildlife Research* **30**(2), 127-133. (doi:http://dx.doi.org/10.1071/WR02042).

4. Gordon G., McGreevy D.G., Lawrie B.C. 1990 Koala population turnover and male social organisation. In *Biology of the Koala* (eds. Lee A.K., Handasyde K.A., Sanson G.D.), pp. 189-192. Chipping Norton, New South Wales, Australia, Surrey Beatty & Sons Ltd.

5. Hasegawa M. 1995 Habitat utilisation by koalas (*Phascolarctos cinereus*) at Point Halloran, Queensland. St. Lucia, Brisbane, Austraia, University of Queensland.

6. Melzer A., Lamb D. 1994 Low density populations of the koala (*Phascolarctos cinereus*) in central Queensland. *Proceedings of the Ryoal Society of Queensland* **104**, 89-93.

7. McGregor D.C., Kerr S.E., Krockenberger A.K. 2013 The distribution and abundance of an island population of koalas (*Phascolarctos cinereus*) in the far north of their geographic range. *PLoS One* **8**(3), e59713. (doi:10.1371/journal.pone.0059713).

8. Hindell M.A. 1984 The feeding ecology of the koala (*Phascolarctos cinereus*) in a mixed *Eucalyptus* forest. Melbourne, Australia, Monash University.

9. Mitchell P., Martin R.W. 1991 The structure and dynamics of koala populations. French Island in perspective. In *Biology of the Koala* (eds. Lee A.K., Handasyde K.A., Sanson G.D.), pp. 97-108. Chipping Norton, New South Wales, Australia, Surrey Beatty & Sons Ltd.

10. Downes S.J., Handasyde K.A., Elgar M.A. 1997 The use of corridors by mammals in fragmented Australian eucalypt forests. *Conservation Biology* **11**(3), 718-726. (doi:10.1046/j.1523-1739.1997.96094.x).

11. Duke T., Masters P. 2005 Confronting a tough issue: fertility control and translocation for over-abundant Koalas on Kangaroo Island, South Australia. *Ecological Management & Restoration* **6**(3), 172-181.

12. Robinson A.C., Spark R., Halstead C. 1989 The distribution and management of the koala (*Phascolarctos cinereus*) in South Australia. *South Australian Naturalist* **64**(1), 4-24.

13. Jurskis V., Potter M. 1997 Koala surveys, ecology and conservation at Eden. (Sydney, Australia, Forest Research and Development Division, State Forests of New South Wales.

14. Gall B.C. 1980 Aspects of the ecology of the koala, *Phascolarctos cinereus* (Goldfuss), in Tucki Tucki Nature Reserve, New South Wales. *Australian Wildlife Research* **7**, 167-176.

15. Faulks J. 1990 The ranging behaviour of koalas. Northern Rivers, New South Wales, Australia, University of New England.
